# Delayed Impact of 2-Oxoadipate Dehydrogenase Inhibition on the Rat Brain Metabolism Is Linked to Protein Glutarylation

**DOI:** 10.3389/fmed.2022.896263

**Published:** 2022-06-01

**Authors:** Alexandra I. Boyko, Irina S. Karlina, Lev G. Zavileyskiy, Vasily A. Aleshin, Artem V. Artiukhov, Thilo Kaehne, Alexander L. Ksenofontov, Sergey I. Ryabov, Anastasia V. Graf, Angela Tramonti, Victoria I. Bunik

**Affiliations:** ^1^Faculty of Bioengineering and Bioinformatics, Lomonosov Moscow State University, Moscow, Russia; ^2^N.V. Sklifosovsky Institute of Clinical Medicine, Sechenov First Moscow State Medical University, Moscow, Russia; ^3^Belozersky Institute of Physico-Chemical Biology, Lomonosov Moscow State University, Moscow, Russia; ^4^Department of Biological Chemistry, Sechenov First Moscow State Medical University, Moscow, Russia; ^5^Institute of Experimental Internal Medicine, Otto von Guericke University Magdeburg, Magdeburg, Germany; ^6^Russian Cardiology Research and Production Complex, Ministry of Health of the Russian Federation, Moscow, Russia; ^7^Faculty of Nano-, Bio-, Informational, Cognitive and Socio-Humanistic Sciences and Technologies, Moscow Institute of Physics and Technology, Moscow, Russia; ^8^Faculty of Biology, Lomonosov Moscow State University, Moscow, Russia; ^9^Institute of Molecular Biology and Pathology, Council of National Research, Department of Biochemical Sciences “A. Rossi Fanelli”, Sapienza University, Rome, Italy

**Keywords:** *DHTKD1*, glutathione, glutarylation, 2-oxoadipate dehydrogenase, citrulline, phosphonate analog of 2-oxoadipate, sirtuin 5

## Abstract

**Background:**

The *DHTKD1*-encoded 2-oxoadipate dehydrogenase (OADH) oxidizes 2-oxoadipate—a common intermediate of the lysine and tryptophan catabolism. The mostly low and cell-specific flux through these pathways, and similar activities of OADH and ubiquitously expressed 2-oxoglutarate dehydrogenase (OGDH), agree with often asymptomatic phenotypes of heterozygous mutations in the *DHTKD1* gene. Nevertheless, OADH/*DHTKD1* are linked to impaired insulin sensitivity, cardiovascular disease risks, and Charcot-Marie-Tooth neuropathy. We hypothesize that systemic significance of OADH relies on its generation of glutaryl residues for protein glutarylation. Using pharmacological inhibition of OADH and the animal model of spinal cord injury (SCI), we explore this hypothesis.

**Methods:**

The weight-drop model of SCI, a single intranasal administration of an OADH-directed inhibitor trimethyl adipoyl phosphonate (TMAP), and quantification of the associated metabolic changes in the rat brain employ established methods.

**Results:**

The TMAP-induced metabolic changes in the brain of the control, laminectomized (LE) and SCI rats are long-term and (patho)physiology-dependent. Increased glutarylation of the brain proteins, proportional to OADH expression in the control and LE rats, represents a long-term consequence of the OADH inhibition. The proportionality suggests autoglutarylation of OADH, supported by our mass-spectrometric identification of glutarylated K155 and K818 in recombinant human OADH. In SCI rats, TMAP increases glutarylation of the brain proteins more than OADH expression, inducing a strong perturbation in the brain glutathione metabolism. The redox metabolism is not perturbed by TMAP in LE animals, where the inhibition of OADH increases expression of deglutarylase sirtuin 5. The results reveal the glutarylation-imposed control of the brain glutathione metabolism. Glutarylation of the ODP2 subunit of pyruvate dehydrogenase complex at K451 is detected in the rat brain, linking the OADH function to the brain glucose oxidation essential for the redox state. Short-term inhibition of OADH by TMAP administration manifests in increased levels of tryptophan and decreased levels of sirtuins 5 and 3 in the brain.

**Conclusion:**

Pharmacological inhibition of OADH affects acylation system of the brain, causing long-term, (patho)physiology-dependent changes in the expression of OADH and sirtuin 5, protein glutarylation and glutathione metabolism. The identified glutarylation of ODP2 subunit of pyruvate dehydrogenase complex provides a molecular mechanism of the OADH association with diabetes.

## Introduction

*DHTKD1*-encoded 2-oxoadipate dehydrogenase (OADH, EC 1.2.4.2) is a recently identified member of the family of the thiamine diphosphate (ThDP)-dependent 2-oxo acid dehydrogenases, found in animals and slime mold *Dictyostelium discoideum* (1). Prediction of the catalytic function of the *DHTKD1* protein as OADH (2) has been supported by increased excretion of the OADH substrate, 2-oxoadipate, and its transamination sibling 2-aminoadipate in urine and blood upon human mutations of *DHTKD1* gene (3–5). 2-Oxo- and 2-aminoadipate are intermediates of the metabolic pathways degrading lysine, hydroxylysine, and tryptophan, in which OADH thus takes part.

The *DHTKD1* mutations in humans mostly lack severe phenotypes, but may be associated with muscle weakness and cardiovascular disease risks (3–7). Some *DHTKD1* variants are enriched in patients with eosinophilic esophagitis (8). Other mutations are shown to cause Charcot-Marie-Tooth disease—a hereditary motor sensory neuropathy, characterized by atrophy of the distal parts of limbs (6, 9, 10). According to a recent study, heterozygous *DHTKD1* variants may also contribute to the phenotype of amyotrophic lateral sclerosis (11). In rare cases, 2-oxoadipate accumulation leads to the vitamin B6-responsive epilepsy, supposed to be caused by toxic reactions with vitamin B6, involving a 2-oxoadipate precursor (5, 12). Metabolic corrections in these patients employing a diet with low lysine and high arginine, decrease the epilepsy markers including 2-oxoadipate, improving the neurological symptoms (13, 14).

Several lines of evidence link the *DHTKD1* expression and/or the OADH substrate 2-oxoadipate to glucose homeostasis. The risk of developing cardiometabolic diseases is increased by elevated levels of 2-aminoadipate (15, 16), while the reduced *DHTKD1* expression in adipose tissue correlates to insulin resistance (17). Accordingly, a higher *DHTKD1* expression increases the insulin sensitivity (18). Our previous study on pharmacological inhibition of OADH in cells suggests that the enzyme function may regulate biosynthesis of nicotinamide adenine dinucleotide (NAD) metabolites from tryptophan, the pathway of high homeostatic significance, particularly for glucose metabolism (19).

Thus, OADH regulation may provide an important therapeutic tool to fight systemic pathologies. However, developing such novel therapeutic approaches requires knowledge of molecular mechanisms of the OADH involvement with (patho)physiological events.

A cellular model of the *DHTKD1* gene silencing shows disturbed mitochondrial function and biogenesis, associated with decreased activity of the tricarboxylic (TCA) cycle enzyme 2-oxoglutarate dehydrogenase (OGDH, EC 1.2.4.2), an isoenzyme of OADH (7, 8, 20). These data are in accord with the known inactivation of OGDH by 2-oxoadipate *in vitro* (21). However, *in vivo*, the 2-oxoadipate concentration in brain does not usually exceed 0.01 mM (3, 22), which is 10–20 times lower than that of 2-oxoglutarate. Hence, unlike the observations in cellular models, significant inactivation of OGDH can hardly be expected *in vivo* even when 2-oxoadipate is increased due to the *DHTKD1* mutation (3, 22). Nevertheless, decreased mitochondrial function and increased reactive oxygen species are observed in epithelial cells of patients with allergic inflammation of esophageal epithelium or Charcot-Marie-Tooth disease, where the *DHTKD1* mutations are enriched (8, 20).

**FIGURE 1:**
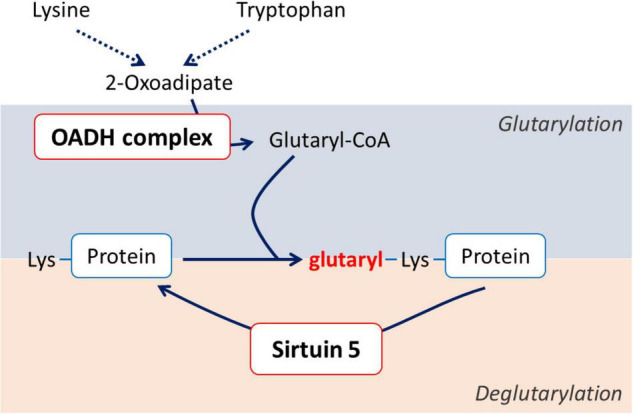
Schematic view of the association between the OADH-catalyzed reaction and protein glutarylation.

In view of the very low concentrations of 2-oxoadipate in most tissues, i.e., small substrate fluxes through OADH, and similar catalytic activities of OADH and the ubiquitously expressed OGDH, we hypothesize that systemic importance of OADH function is linked to the enzyme participation in homeostatic regulation through post-translational modifications of proteins. As a producer of reactive glutaryl residues, either ThDP-bound in the isolated OADH, or CoA-bound in its multienzyme complex (OADHC), OADH may be a part of the system of post-translational protein modification by glutarylation (Figure 1). Like other acyl-CoA’s, glutaryl-CoA may modify lysine residues of proteins (23). The accompanying change in the lysine residue charge from the positive to negative one may be involved in functional regulation of glutarylated proteins (23, 24). Removal of glutaryl moieties is performed by specific deacylase of the negatively charged acyl groups—NAD^+^-dependent deglutarylase/desuccinylase/demalonylase sirtuin 5 (EC 2.3.1.B43). Sirtuin 5 has neuroprotective significance under ischemic conditions in the brain (25). In the model of spinal cord injury (SCI) the protein expression of sirtuin 5 correlates positively with the rehabilitation of animals (26). On the contrary, excessive protein glutarylation upon increased levels of glutaryl-CoA is known to cause neurological disorders (27). Such neurological significance of protein glutarylation implies that the balance of activities of the glutaryl-CoA producer OADH and protein deglutarylase sirtuin 5 may be an important homeostatic determinant in neural tissue.

The goal of our current work is to experimentally test the hypothesis that systemic significance of the function and expression of OADH is related to the enzyme participation in protein glutarylation. Taking into account delayed effects of metabolic alterations in neuropathologies, that may be mediated by post-translational acylation of proteins, mostly studied regarding acetylation of histones (26, 28–33), we characterize long-term changes in protein glutarylation, OADH expression and metabolism, induced by a short-term pharmacological challenge of OADH function in animals of different (patho)physiological states. Our choice of the pathology model for these studies accounts for several considerations. As noted above, the *DHTKD1* mutations are often associated with muscle weakness (4, 5) and Charcot-Marie-Tooth disease (6, 9, 10). It is remarkable in this regard that our search on the *DHTKD1* expression in Gene Expression Omnibus database^1^ has revealed that the SCI at T8 vertebra, known to induce muscle atrophy due to impaired muscle innervation, is associated with changed *DHTKD1* expression, both in the injured spinal cord and skeletal muscles (Supplementary Figure 1). In the current study we therefore use a rat model of SCI and specific OADH-directed inhibitor adipoyl phosphonate (19, 34) in its membrane-permeable trimethylated form (trimethyl ester of adipoyl phosphonate, TMAP) to investigate (patho)physiological significance of OADH function and its link to glutarylation in neural tissue. Based on previous studies on systemic significance of metabolic changes in cerebral cortex (26, 31, 35–38), we select this brain region to reveal the consequences of the perturbed OADH function for the brain metabolism in healthy and diseased animals. Addressing the goals of current study using this tissue also considers neurological outcome of the *DHTKD1* mutations (3, 6, 9–11) and significant changes in cellular metabolism, including that of the deacylases substrate NAD^+^, upon the OADH inhibition in cells where the enzyme expression is low, as also observed in the brain cortex (19).

## Materials and Methods

### Materials

All used reagents were of the highest purity grade available. Trimethyl ester of adipoyl phosphonate (TMAP) was synthesized according to (19). EDTA was purchased from Serva (Germany); methanol—from Merck (Germany); Triton-X 100, KH_2_PO_4_, and NaCl—from Panreac (Spain); glycerol—from MP Biomedicals, LLC (Santa Ana, CA, United States). NAD^+^ was obtained from Gerbu (Heidelberg, Germany), oxidized glutathione—from Calbiochem (La Jolla, CA, United States). All other reagents were of the highest purity available and obtained from Sigma-Aldrich (Helicon, Moscow, Russia). Deionized MQ-grade water was used to prepare solutions. The used antibodies are indicated in section “Western-Blotting Quantification of the Protein Levels of OADH, Sirtuin 3, and Sirtuin 5 and of Glutarylated Proteins in Rat Cerebral Cortex.”

### Animal Husbandry

Manipulations with rats were carried out in accordance with the international recommendations of Good Laboratory Practice (GLP), methodical recommendations for laboratory animal care (Agricultural-Industrial Guidance Document 3.10.07.02-09), European Convention for the Protection of Vertebrate Animals Used for Experimental and Other Scientific Purposes, Strasbourg, 1986 ETS No. 123), as well as Guidelines for accommodation and care of animals, including species-specific provisions for laboratory rodents and rabbits developed by Rus-LASA (No. 33216-2014, 01.07.2016) and internal rules of Russian Cardiology Research and Production Complex. The experimental protocols were approved by Bioethics Committee of Russian Cardiology Research and Production Complex (Protocol No. 3, 23.03.2016) and Bioethics Committee of Lomonosov Moscow State University (protocol number 69-o from 09.06.2016). The study was not pre-registered. The minimum necessary size of the animal sample was estimated by *t*-test using a power of 80% and a level of significance of 0.05.

The study was exploratory, and no exclusion criteria were pre-determined. The animals were kept in standard conditions with 12 h light and 12 h dark cycle in individual cages with free access to water and meal.

### Spinal Cord Injury Model and Administration of the 2-Oxoadipate Dehydrogenase Inhibitor

**FIGURE 2:**
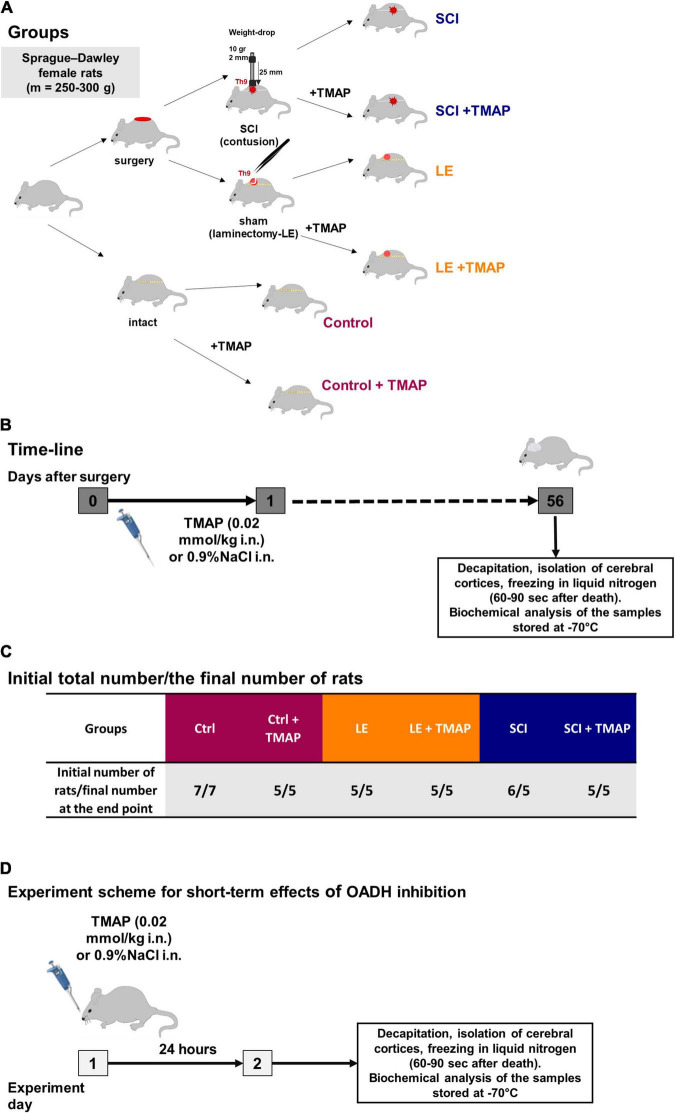
Experimental flow-chart. **(A)** An overview of experimental procedures for each animal group in SCI model. **(B)** Timeline of the procedures in SCI model. **(C)** Distribution of rats across the groups in SCI model with initial and end point number of animals in each group. **(D)** Timeline of the procedures in the study on short-term effects of the OADH-directed inhibitor TMAP. The color code of the experimental groups, given in **(A,C)**, is employed in the following presentation of the results in different figures.

The SCI model and postsurgical care were described in details in previous works (26, 33). Severe SCI was performed using the weight-drop method that allows maximal standardization of the injury level (39). The rats were purchased from Nursery of laboratory animals, Institute of Bioorganic Chemistry (Pushchino, Russia). The adult female Sprague–Dawley of 12–13 weeks (weighing 230 ± 20 g) were exposed to laminectomy (LE) or SCI at the T9 vertebra, with their follow-up ended after 8 weeks, i.e., at the corresponding age of 20–21 weeks (weighing 290 ± 20 g in LE group and 265 ± 15 g in SCI group) (Figure 2B). Sham-operated animals were subjected to LE without affecting the dura matter. LE was associated with the formation of granuloma, affecting muscles, vessels, and connective tissue at the site of the operation.

Intranasal administration of a water solution of TMAP (at a dose of 0.02 mmol/kg) were performed once in the morning following the operation, i.e., within 15–20 h after the operation. This experimental design imitated potential therapeutic intervention after the neurotrauma. Intranasal application was used as a non-invasive method providing an access to the CNS for different molecules that do not cross the blood-brain barrier (40). Control animals received the corresponding administration of physiological solution (0.9% NaCl). No nasal bleeding was observed. In total, 33 rats were involved in the SCI model study. One rat died during the post-surgical recovery period. The resulting 32 rats were distributed among the experimental groups as shown in Figures 2A,C.

Eight weeks after the operations, the rats were decapitated, the brains were excised and transferred on ice. The cerebral hemispheres (called as cerebral cortex further in the text) were separated from other brain parts and frozen in liquid nitrogen 60–90 s after decapitation. The cortices were stored at −70°C before biochemical analyses.

### Independent Experiments on Short-Term Effects of Administration of the 2-Oxoadipate Dehydrogenase Inhibitor

Intranasal administration of TMAP was also performed in the study of the short-term consequences of the TMAP treatment (Figure 2D), using the male Wistar rats obtained from the Russian Federation State Research Center Institute of Biomedical Problems RAS (IBMP) (265 ± 10 g, 8–10 weeks old). The TMAP administration was as described in section “SCI Model and Administration of the OADH Inhibitor.” The rats were sacrificed by decapitation using a guillotine (OpenScience, Russia) 24 h after the administration of the OADH inhibitor. The brain cortices were excised and frozen as described above. No rats died or were excluded during the short-term experiment.

### Homogenization and Extraction of Rat Tissues

To assay the enzymatic activities, halves of the cortex tissue were homogenized according to the previously published protocol (41). Homogenization buffer contained 50 mM MOPS pH 7.0, 2.7 mM EDTA, 20% glycerol and the mammalian protease inhibitors cocktail. For metabolic profiling, another half of the brain cortex was extracted with methanol and acetic acid according to the published procedure (33, 42).

### Enzymatic Assays

Extramitochondrial activity of multienzyme OADH complex (OADHC), designated as OADHCem in the further text, and activities of enzymes of central carbon metabolism were measured in the brain homogenates as described in (26, 41, 43), using Sunrise microplate reader (Tecan, Grödig, Austria). The multienzyme assay scheme ensured at least three technical replicates for each sample. Activities of enzymes are expressed in μmol of a product generated per min per g of the tissue fresh weight (FW). The enzymatic activities were measured at saturating concentrations of all the substrates and cofactors. The maximal reaction rate of an enzyme or enzymatic complex was thus estimated, corresponding to the functional expression of an enzyme or its multienzyme complex in the tissue homogenate.

### Western-Blotting Quantification of the Protein Levels of 2-Oxoadipate Dehydrogenase, Sirtuin 3, and Sirtuin 5 and of Glutarylated Proteins in Rat Cerebral Cortex

The levels of sirtuin 3 (EC 2.3.1.286), sirtuin 5, OADH and protein glutarylation were estimated by western-blotting using primary antibodies from Cell Signaling Technology (Danvers, MA, United States) #8782 and #5490 for sirtuin 5 and sirtuin 3, respectively, Thermo Fisher Scientific (Waltham, MA, United States) #PA5-24208 for OADH and PTM Biolabs (Chicago, IL, United States) #PTM-1151 for glutaryllysine. The primary antibodies for sirtuin 5, sirtuin 3, OADH protein and glutaryllysines were used in 1:1,000, 1:2,000, 1:200 and 1:2,000 dilutions, respectively, with the appropriate secondary anti-rabbit HRP-conjugated antibodies from Cell Signaling Technology, #7074. The relative quantification of chemiluminescence was performed in ChemiDoc Imager (Bio-Rad, Hercules, CA, United States) and Image Lab software version 6.0.1 (Bio-Rad, Hercules, CA, United States). Normalization of the protein levels to the total protein in the corresponding gel lanes was performed using the protein fluorescent quantification with 2,2,2-trichloroethanol, similarly to the published procedure (44). The band intensities from different membranes were compared across all the membranes after the normalization on the levels of the common samples repeated on independent membranes.

### Metabolic Profiling of the Rat Brain Extracts

Amino acids and related compounds were quantified in extracts of cerebral cortices according to (26, 33, 42) using the amino acid analyzer L-8800 (Hitachi Ltd., Japan), employing a gradient of Li-citrate buffers and the ninhydrin reagent (Wako Pure Chemical Industries; P/N 298-69601). Glutathione disulphide (GSSG) was quantified using fluorescence of its product with o-phthalic aldehyde according to the method described in (45) and optimized in (46). Tryptophan levels in the brain extracts were determined as described in (47) with modifications according to (48), using the tryptophan conversion into fluorescent norharman. The fluorescent signal was obtained at λ_*ex*_/ λ_*em*_ of 365/460 nm. NAD^+^ levels in the brain extracts were measured as described in (49).

### Mass-Spectrometric Detection of Glutarylation

Mass-spectrometric detection of ODP2 (EC 2.3.1.12) glutarylation was carried out in the cerebral cortices of male Wistar rats. The tissue samples were treated according to the previously published protocol (50) and subjected for SDS-PAGE electrophoresis with the concentration of the separating gel of 10% (51). Gel lanes from 25 to 75 kDa were excised, subjected to proteolysis by trypsin, and the resulting peptide fragments were analyzed by LC-MS with detection of modified and unmodified peptides according to the previously published protocol (50). The peptides for quantification of the ODP2 glutarylation level are given in Table 1.

**TABLE 1:** Characterization of ODP2 peptides.

Peptide	Specification	C13-isotopomeric variants of the precursors		
		
		monoisotopic	[M+1]	[M+2]
KELNK(+114.03) MLEGK	K451 glutarylated	652.3499++	652.8514++	653.3521++
GLETIASDVVSLASK	normalization peptide	745.409++	745.9105++	746.4119++

*The peptides are used for the relative quantification of ODP2 K451 glutarylation by MS in the rat brain cortex. Several precursor variants with monoisotopic mass and the C13-isotopomeric variants ([M+1] and [M+2]) have been detected to increase the quantification accuracy.*

Mass-spectrometric detection of OADH glutarylation was performed using the recombinant human OADH expressed in *Pichia pastoris* and purified according to the previously published protocol (1). The major protein band of 100 kDa after SDS-PAGE electrophoresis with the concentration of the separating gel of 10% was analyzed by LC-MS as above.

### Statistical Analysis

All data were analyzed using GraphPad Prism 7.0 software (GraphPad Software, Inc., La Jolla, CA, United States) or RStudio^2^ and shown as mean ± SEM. For metabolic profiling visualization and correlation analysis *pheatmap* and *ggstatplot* R packages were used, respectively. Changes in specific biochemical parameters are presented as box-and-whisker plots, showing quantiles of each sample distribution. For comparison of more than two groups, two-way analysis of variance (ANOVA) and *post-hoc* Tukey’s test were used. The two-tailed *p*-values 0.05 were considered to indicate statistically significant differences, shown in the figures.

## Results

### Analysis of Overall Metabolic Impact of 2-Oxoadipate Dehydrogenase Inhibition Under Different (Patho)physiological Conditions

**FIGURE 3:**
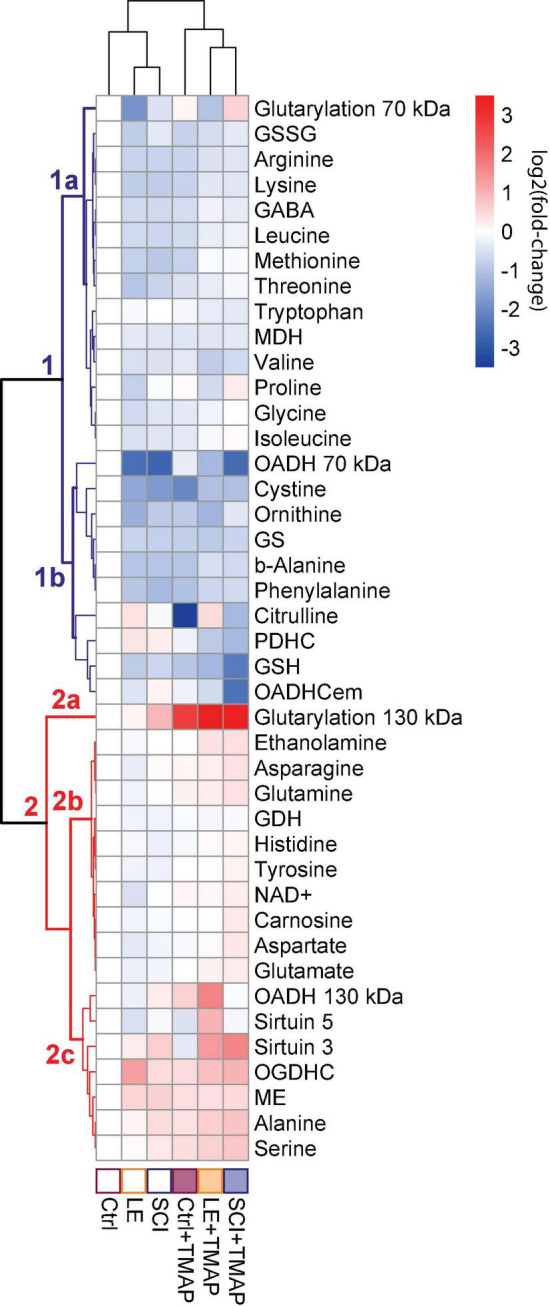
Clusters of metabolic changes induced by the OADH-directed inhibitor TMAP in the cerebral cortices of the control (Ctrl), sham-operated (LE) and SCI rats. Metabolic changes in the cerebral cortex are presented as log_2_(fold-change), where a fold-change is a ratio of the mean metabolite level in the treated group to the corresponding mean level in the control group. These normalized values are shown in the heatmap according to the color code of the scale bar. The animal groups are indicated at the bottom of the heatmap and assigned the color code used in all the figures presenting the data on these groups. Metabolites (at the right side of the heatmap) and experimental groups (at the bottom of the heatmap) are clustered using ward.D method from *pheatmap* package in RStudio. The main metabolic clusters are numbered 1 and 2, each having the subclusters designated by the letters. The cluster 1 with decreased levels of metabolites is assigned the blue color, while the cluster 2 with increased levels of metabolites is shown in red. Number of animals in each group: Ctrl, *n* = 7; LE, *n* = 5; SCI, *n* = 5; Ctrl + TMAP, *n* = 5; LE + TMAP, *n* = 5; SCI + TMAP, *n* = 5.

To identify the pathways associated with OADH function in the brain, where OADH/*DHTKD1* expression is relatively low (19), the metabolism of the rat cerebral cortex has been challenged by specific OADH inhibitor TMAP in different (patho)physiological states of the rats. Based on an earlier finding of the long-term time-dependent biphasic changes in the *DHTKD1* expression following SCI (Supplementary Figure 1), the metabolic changes 8 weeks after a single administration of TMAP have been determined in the female rats of the control, LE and SCI groups (Figure 3). The usual levels of the OADH substrate 2-oxoadipate and its transamination sibling 2-aminoadipate are far too low for unambiguous detection of their changes in animal tissues. However, the amino acid metabolism, where OADH takes part, is tightly interconnected. Therefore, the amino acid profiles in the rat cerebral cortex are used as indicators of OADH function. To analyze the associated changes in the functional expression of the enzymes of amino acid metabolism, the activities of such enzymes are included in the metabolic cluster maps. To assay the OADH and OGDH reactions, catalyzed by the enzymes assembled into the multienzyme complexes (OADHC and OGDHC, respectively), the previously elaborated protocol is followed (41). The extramitochondrial activity of OADHC (OADHCem) is assayed before the mitochondrial solubilization that releases the intramitochondrial activities of both the OGDH and OADH complexes. The intramitochondrial activity of OADHC cannot be discriminated from a much higher level of the activity of OGDHC, also catalyzing the reaction with 2-oxoadipate. However, because of its low expression, OADHC does not significantly contribute to the assayed intramitochondrial OGDHC activity. In view of the problems with assaying the intramitochondrial activity of OADH, where major part of the enzyme is supposed to be localized, the expression of the previously identified OADH isoforms of 70 and 130 kDa (1) is determined by western-blotting. Finally, given the possible link between the OADH-dependent production of glutaryl residues and system of post-translational modification of proteins by glutarylation (Figure 1), the levels of glutarylated proteins and deglutarylase sirtuin 5 in the brain are also included in the metabolic heatmap. Expression of the major mitochondrial deacetylase sirtuin 3 is determined in view of its involvement into the regulation of mitochondrial metabolism, particularly the enzymes of the amino acid metabolism (50, 52).

Overall, the changes in 42 biochemical parameters of the brain, quantified 8 weeks after a single TMAP administration to the control, LE or SCI rats, as well as the changes due to LE and SCI, all relative to the control levels, are shown as a heatmap in Figure 3. Different sets of biochemical parameters that change in concert, form clusters shown as a tree at the left of the heatmap, while the level of similarity between specific metabolic states is shown in the tree above the heatmap (Figure 3). Thus, the clustering procedure reveals certain sets of linked biochemical parameters, as well as specific and common features of the analyzed (patho)physiological states.

The clustering tree of the metabolic states (Figure 3, clusters above the heatmap) shows that TMAP administration is the main differentiator between the studied animal groups, as all the groups treated with TMAP are clearly separated from the rats not exposed to TMAP, independently of their (patho)physiological state. The clustering tree of metabolites (Figure 3, clusters at the left of the heatmap) points to the two major clusters of the first level, each headed by the glutarylated proteins of 70 and 130 kDa, respectively. Glutarylated proteins of 70 kDa are associated with the cluster 1 combining parameters which mostly decrease after OADH inhibition in different (patho)physiological states, as compared to those in the control rats. This “blue” cluster 1 includes subclusters of the less (above, 1a) and more (below, 1b) strong changes. The stronger changes include the expression of OADH 70 kDa, activity of OADHCem, activity of PDHC and levels of a number of redox metabolites, such as glutathione, its precursor cystine, and citrulline—a marker of NO synthesis from arginine. Glutarylated proteins of 130 kDa (branch 2a at Figure 3), representing the key long-term effect of TMAP administration across all the three studied states (control, LE and SCI), are associated with the “red” cluster 2, combining parameters which mostly increase, as compared to those in the control rats. The “red” cluster is further divided into the subclusters of the parameters changing less (above, 2b) and more (below, 2c) across the studied groups in comparison to their levels in the control rat brain. The stronger changes in the subcluster 2c are inherent in the expression of OADH 130 kDa, deglutarylase sirtuin 5, deacetylase sirtuin 3, activities of OGDHC and NADP^+^-dependent malic enzyme, as well as the levels of the amino acids serine and alanine (Figure 3).

### Pyruvate Dehydrogenase Complex Glutarylation

**FIGURE 4:**
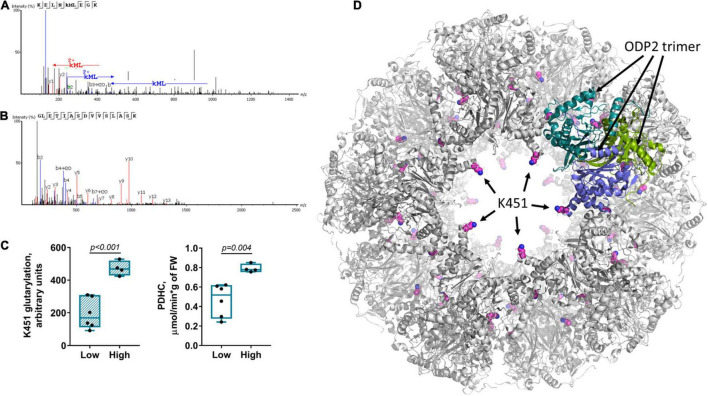
Glutarylation of pyruvate dehydrogenase complex. The glutarylation of lysine K451 of the second component of rat PDHC (ODP2 protein, encoded by *DLAT* gene) is identified in the rat brain by LC-MS. **(A)** The spectra of the peptide with K451 glutarylation. **(B)** The spectra of non-glutarylated peptide, used for normalization. **(C)** Correspondence of K451 glutarylation of ODP2 to PDHC activity: The six animals with the low K451 glutarylation level also have the low PDHC activity, while the four animals with the high glutarylation level have the high PDHC activity. The animal subgroups exhibit significant differences in the parameters, determined by unpaired Mann-Whitney test and shown as *p*-values in the graphs. **(D)** Localization of the glutarylated residue (shown with pink carbon atoms) in the ODP2-formed core of PDHC, according to the structure (PDB: 6CT0) of human protein, whose K466 corresponds to K451 of the rat protein. Subunits of one of the ODP2 trimers, organized by five around the “pore” to the inner cavity of the core, are colored in green, blue and marine. As seen from the structure, K451/K466 residue faces the pore. The residue glutarylation would change electrostatics of the entrance to/exit from the inner cavity of the core.

It is remarkable that the subcluster 1b within the “blue” cluster 1, which is headed by the glutarylated proteins of 70 kDa, comprises changes in the activity of PDHC, levels of the redox-state-related metabolites, such as glutathione and citrulline, along with changes in expression of OADH 70 kDa and activity of OADHCem (Figure 3). While the link of PDHC activity to the brain redox state is well-known, the clustering of these indicators with the expression and activity of OADH suggests the PDHC regulation by glutarylation. In fact, the second enzymatic component of PDHC, dihydrolipoamide acetyltransferase (ODP2, encoded by *DLAT* gene) has the same molecular mass (∼70 kDa) as one of the two major bands of the brain glutarylated proteins (Supplementary Figure 2). In view of the known proteolysis of this protein, and potential glutarylation of other PDHC components, we have identified the glutarylated proteins of 25–75 kDa in the homogenates of the rat brain cortex by mass-spectrometric analysis. Glutarylation of the brain ODP2 subunit of PDHC at K451 residue is revealed in this experiment. The corresponding MS/MS spectra of the glutarylated ODP2 peptide are shown in Figure 4A.

Quantification of the level of ODP2 glutarylated peptide in a sample of control rats is based on normalization of the glutarylated peptide abundance to ODP2 expression, estimated by simultaneous quantification of the well-defined ODP2 peptide shown in Figure 4B. This quantification reveals a high interindividual variability in the levels of glutarylation of ODP2, that interferes with identification of statistically significant differences in this parameter upon comparison of different animal groups. Among the factors potentially affecting the variability, we have assessed its response to such known metabolic regulators of the brain metabolism as vitamins B1 and B6. The animals supplemented with 100 mg per kg of vitamins B1 and B6 as described earlier (43), do not exhibit any shift in the ODP2 glutarylation level, compared to the control animals. In both cases, the two different subgroups of the animals could be seen. Those with the low ODP2 glutarylation level also possess the low PDHC activity, while the animals with the high glutarylation level have high PDHC activity. The differences between these subgroups become statistically significant in the pooled sample of the control and vitamin-supplemented animals (Figure 4C). The data suggest regulatory role of K451 glutarylation of ODP2 in PDHC.

Structural analysis reveals that the glutarylated K451, belonging to the catalytic domain of ODP2, extends into the pores of the dodecahedral 60-meric core of mammalian PDHC (Figure 4D). As the pores may be important for the CoA entry to the catalytic channel of ODP2 from the inner cavity of the core (53), our analysis favors functional importance of ODP2 glutarylation. As a result, the OADH-dependent glutarylation of PDHC may underlie the common allocation of the PDHC activity, redox-related metabolites, OADH 70 kDa isoform expression and OADHCem activity within the subcluster 1b of the “blue” cluster 1 headed by glutarylated proteins of 70 kDa.

### Long-Term Changes in the 2-Oxoadipate Dehydrogenase-Associated Parameters of the Rat Cerebral Cortex

**FIGURE 5:**
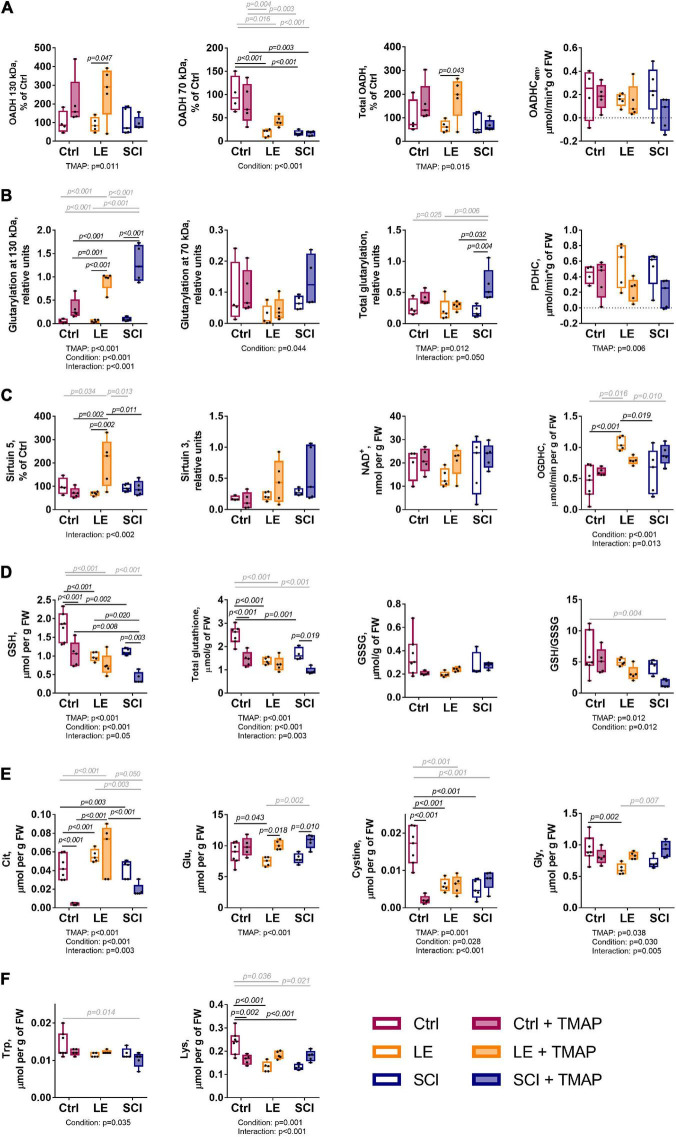
Long-term changes in selected biochemical parameters of the cerebral cortex of the control, LE and SCI rats 8 weeks after a single administration of the OADH-directed inhibitor TMAP. Representative Western blots for relative glutarylation and protein levels are shown in Supplementary Figures 2–5. **(A)** Relative expression of the OADH isoforms 130 and 70 kDa, total OADH and enzymatic activity of extramitochondrial OADHC. **(B)** Relative glutarylation of the brain proteins of 130 and 70 kDa, total glutarylated protein and PDHC activity. **(C)** Expression of sirtuin 5, sirtuin 3, level of NAD^+^ and enzymatic activity of OGDHC. **(D)** Glutathione redox state homeostasis: levels of reduced glutathione (GSH), total glutathione, oxidized glutathione (GSSG), and the glutathione redox ratio. **(E)** Levels of metabolites involved in glutathione homeostasis: citrulline (Cit), cystine, glutamate (Glu), glycine (Gly). **(F)** Metabolic indicators of the OADH function: tryptophan (Trp) and lysine (Lys). Statistical significance of differences between experimental groups is determined by two-way ANOVA with Tukey’s *post-hoc* test. Factor significances and their interaction, estimated by ANOVA, are shown as *p*-values (*p* < 0.05 only) below the graphs. The *p*-values on the graphs show the results of the *post-hoc* test, that are in black or gray for the experimental groups differing by one or two factors, correspondingly.

Statistically significant differences between the studied groups have been assessed for selected metabolic parameters (Figure 5) from the OADH-comprising subclusters (Figure 3). The three indicators of OADH function, i.e., the extramitochondrial activity of OADHCem and the protein expression of the two OADH isoforms, respond differently to the interventions (Figure 5A). Expression of 130 kDa isoform of OADH is influenced by the TMAP treatment (*p* = 0.011 for the TMAP factor), significantly elevated in the TMAP-treated LE animals compared to LE rats without TMAP (*p* = 0.047). In contrast, 70 kDa isoform of OADH is significantly affected by the (patho)physiological state of animals (*p* = 0.001 for the condition factor). That is, independent of the TMAP treatment, either LE or SCI animals exhibit a lower level of 70 kDa OADH than is inherent in the control animals (Figure 5A). The OADHCem activity does not exhibit significant changes across the studied conditions.

Regarding glutarylation of the brain proteins of 130 kDa, both the TMAP administration and (patho)physiological state are significant and interacting factors, based on the ANOVA analysis provided under the graphs in Figure 5B. In contrast, only the animal state significantly affects glutarylation of 70 kDa proteins. Comparison of the studied groups indicates that the TMAP administration significantly increases glutarylation of 130 kDa proteins in both the LE and SCI animals, but not in the control animals. The total glutarylation is increased by TMAP only in SCI animals (Figure 5B).

The TMAP effect on the sirtuin 5 expression (Figure 5C) reciprocates the TMAP-induced increase in total glutarylation (Figure 5B). That is, there is an increase in sirtuin 5 expression by TMAP in LE, but not in SCI animals. In contrast, the total glutarylation increases in SCI animals, but is constant in LE animals. These findings indicate that a long-term effect of the OADH-directed inhibitor on the brain protein glutarylation is tightly linked to the regulation of the sirtuin 5 expression, with the regulation depending on pathophysiological state of the animals.

**FIGURE 6:**
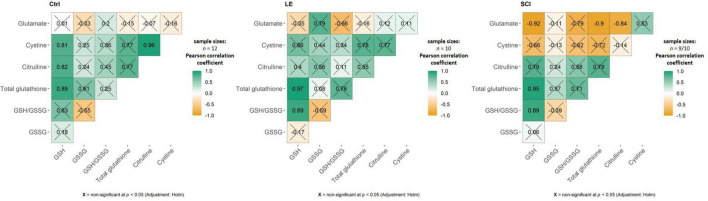
Pearson’s correlation matrices between the brain metabolites involved in glutathione biosynthesis. Each of the three shown groups, i.e., the control, LE and SCI rats, include animals with and without administration of the OADH-directed inhibitor TMAP. Sample sizes for the estimated metabolites are indicated in the figure. In SCI, sample with *n* = 9 refers to the GSSG level estimation. Non-significant (*p* ≥ 0.05) correlations after Holm-Bonferroni multiple testing adjustment are crossed out. The figure is created using *ggstatsplot* package in Rstudio.

As shown in Figure 5D, the TMAP exposure strongly diminishes the brain glutathione levels in the control and SCI animals, but not in LE animals, where expression of sirtuin 5 is increased. Thus, the TMAP-induced perturbation impairs glutathione homeostasis when the perturbation is not addressed by an increase in the sirtuin 5 expression. However, the mechanisms of the TMAP-induced decreases in the glutathione level in the control and SCI brains are different. This is obvious from the associated changes in the levels of the glutathione precursors—cystine, glutamate, glycine—and the level of the marker of NO⋅ production citrulline (Figure 5E). In the control animals, the glutathione decrease is accompanied by the decrease in the cystine level (Figures 5D vs. 5E). Simultaneous decline in the level of citrulline (Figure 5E) points to insufficient generation of the cystine transporter activator NO⋅ and diminished cystine transport (54). However, neither cystine, nor citrulline levels are significantly reduced by the TMAP treatment of SCI animals, suggesting another mechanism of the glutathione drop after SCI. Among these mechanisms, OGDHC dysfunction is a factor that may perturb the redox status of the brain glutathione buffer (55). Yet in our experimental settings, the TMAP treatment is not significant for the OGDHC activity, which responds to the (patho)physiological state only (Figure 5C, condition significance). From the parameters analyzed, the increased glutarylation in SCI animals coincides with the TMAP-induced decrease in the brain glutathione levels (Figures 5B vs. 5D). The role of glutarylation in the glutathione homeostasis is supported by the fact that in LE animals, neither the glutathione homeostasis nor the glutarylation are perturbed by TMAP, as the inhibitor administration increases the sirtuin 5 expression. The different homeostatic mechanisms maintaining the brain glutathione level in response to the TMAP treatment in different (patho)physiological states, are further supported by the correlation analysis of the three experimental groups comprising the animals with and without TMAP (Figure 6). In control animals, cystine and citrulline levels are strongly correlated to each other and to the levels of reduced glutathione, that is in accordance with the NO^⋅^-dependent cystine supply being limiting for glutathione biosynthesis. In SCI animals, these strong correlations disappear, while the reduced glutathione becomes inversely correlated to the level of its precursor glutamate.

The levels of OADH-related amino acids lysine and tryptophan (Figure 1) decrease in the LE and/or SCI animals, compared to the control animals. These decreases depend on the (patho)physiological state of the animals more than on the TMAP administration (Figure 5F). The lysine levels show a significant interaction between the animal state and TMAP administration, while the levels of tryptophan decrease significantly in the TMAP-treated SCI animals compared to the control ones (Figure 5F).

### Concordance Between the 2-Oxoadipate Dehydrogenase Expression and Brain Protein Glutarylation

**FIGURE 7:**
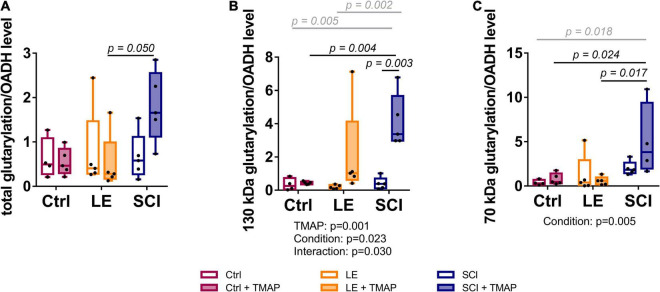
Levels of brain protein glutarylation, normalized to OADH expression, in the rat cerebral cortex of the studied animal groups. Representative Western blots for estimation of relative glutarylation and OADH levels are shown in Supplementary Figures 2, 3, respectively. **(A)** Total protein glutarylation levels normalized to the sum of the expression of the two OADH isoforms. **(B)** Levels of glutarylation of 130 kDa proteins normalized to expression of 130 kDa isoform of OADH. **(C)** Levels of glutarylation of 70 kDa proteins normalized to expression of 70 kDa isoform of OADH. Statistically significant (*p* < 0.05) differences between experimental groups, determined by two-way ANOVA with Tukey’s *post-hoc* test, are shown on the graphs. The black and gray colors correspond to the groups differing in one or two factors, respectively. Statistically significant (*p* < 0.05) factors and their interactions, estimated by ANOVA, are shown as *p*-values below the graphs.

As revealed by Western-blotting (Supplementary Figures 2, 3), the major glutarylated proteins and OADH isoforms in the brain have the same molecular masses, i.e., 130 and 70 kDa. Together with the known autoacylation reactions in the 2-oxo acid dehydrogenases (53), this finding suggests that the TMAP-induced increases in both the OADH expression (Figure 5A) and brain protein glutarylation (Figure 5B) manifests autoglutarylation of OADH isoforms, as occurrence of this side reaction is expectedly proportional to the OADH expression. The ratios of the glutarylated proteins to the OADH expression have therefore been calculated for each animal (Figure 7). Such analysis shows that the TMAP effects on the brain glutarylation of 130 kDa, 70 kDa and/or total proteins in the control and LE animals disappear when normalized to the OADH expression (Figure 7, control and LE rats). In contrast, in the TMAP-treated SCI rats, also the normalized protein glutarylation exhibits an increase, compared to the non-treated SCI animals (Figure 7, SCI rats). The increase is statistically significant regarding glutarylation of 130 kDa proteins (Figure 7, SCI in the middle panel). Besides, statistically significant differences in the normalized glutarylation of total proteins and 70 kDa proteins are observed between the TMAP-treated animals after SCI and LE (Figure 7, the left and right panels). Thus, only in SCI animals, the normalized glutarylation of brain proteins after the short-term OADH inhibition exceeds the glutarylation proportional to the OADH expression.

**FIGURE 8:**
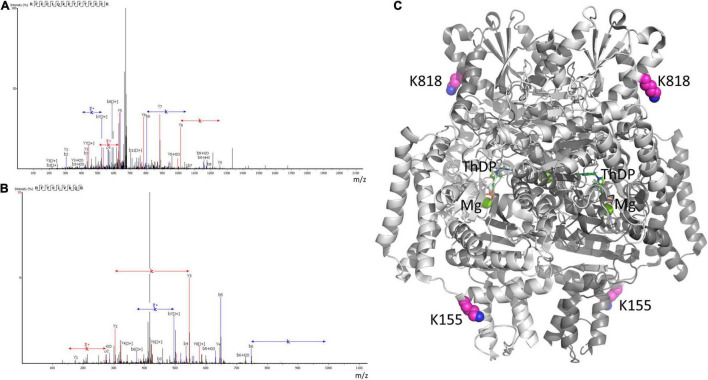
Mass-spectrometric identification of OADH glutarylation. The glutarylation of lysine K155 and K818 is identified by LC-MS in human recombinant OADH expressed in yeast. **(A)** The spectra of the peptide with glutarylated K155, namely RFEELQK(+114.03)ETFTTEER, m/z = 686.3347, retention time (RT) = 96.49; **(B)** the spectra of the peptide with glutarylated K818, namely HFYSLVK(+114.03)QR, m/z = 431.2349, RT = 82.38; **(C)** localization of the identified glutarylated residues (the carbon atoms marked in pink) in the OADH dimer. The available structure of the enzyme dimer crystallized with Mg^2+^ThDP (PDB: 6SY1) is shown, with the active site regions defined by ThDP and magnesium ions (shown in green).

In view of the low OADH expression in the brain, the mass-spectrometric identification of the enzyme peptides in the tissue homogenates is below the detection limit. However, glutarylation of OADH is confirmed by our mass-spectrometry analysis of human OADH protein, overexpressed in yeast. In the purified recombinant OADH the peptides with glutarylated OADH residues K155 and K818 are determined (Figures 8A,B). Structural analysis shown in Figure 8C reveals that the glutarylated residues are located on the protein surface away from the active sites.

### Short-Term Changes in Glutarylation System Upon 2-Oxoadipate Dehydrogenase Inhibition

**FIGURE 9:**
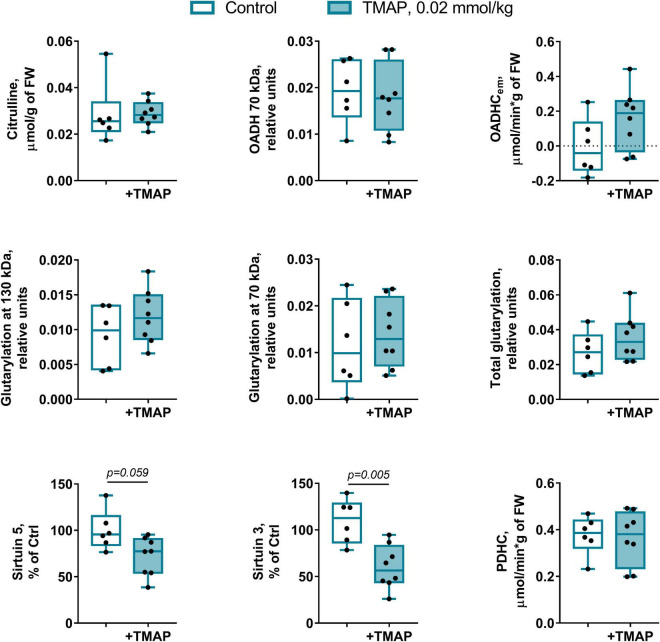
Short-term changes in the protein glutarylation system of the brain cortex 24 h after TMAP administration to the control rats. Representative Western blots for estimation of relative glutarylation and protein levels are shown in Supplementary Figure 6. Statistical significance of the differences between experimental groups is determined by unpaired Mann-Whitney test, with *p*-values < 0.10 shown on the graphs.

**FIGURE 10:**
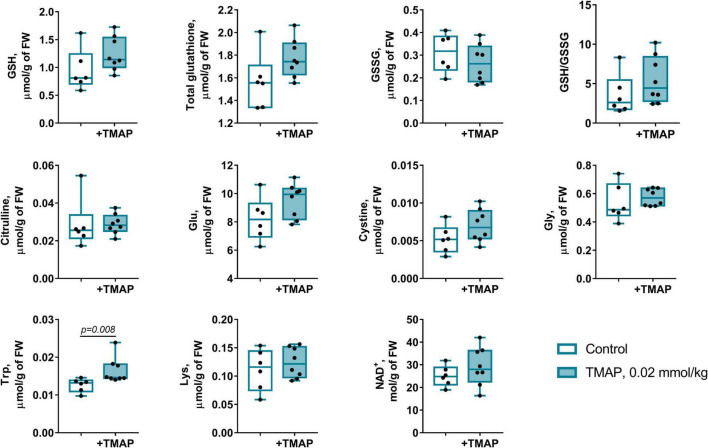
Short-term changes in the redox-state- and OADH-related metabolites in the brain cortex 24 h after a single administration of TMAP. Statistically significant (*p* ≤ 0.05) differences between experimental groups are determined by unpaired Mann-Whitney test, with *p*-values shown on the graphs.

The long-term metabolic consequences of a single TMAP administration cannot be caused by the permanent action of a water-soluble inhibitor during 8 weeks, as such inhibitors are usually excreted within 24 h. It thus appears that the observed long-term consequences for metabolism (Figures 3, 5) result from the short-term perturbation of the brain glutarylation system by administration of TMAP. To support this assumption, we have assessed the short-term effects of OADH inhibition, estimating key parameters of the brain glutarylation system (Figure 9) and metabolism (Figure 10) 24 h after the TMAP administration to control animals. Within this time period, prompt responses of the brain metabolism to different challenges including drug administration, are observed, but the time is not enough for metabolic reprogramming (56, 57). Indeed, no significant changes in the brain OADH expression or protein glutarylation are observed in this case (Figure 9). However, the TMAP-induced inhibition of the glutaryl-CoA producer OADHC is accompanied by decreased levels of the deglutarylase sirtuin 5 and deacetylase sirtuin 3 (Figure 9), linking the OADH function to the brain protein acylation. These short-term changes are not accompanied by metabolic perturbations observed upon the long-term changes in the glutarylation system. Among the metabolites tested in both the long-term and short-term experiments, the only significant short-term effect of TMAP is an increase in tryptophan (Figure 10), that is in good accord with the OADH participation in tryptophan degradation. Thus, the short-term consequences of the TMAP administration are an increase in the level of tryptophan manifesting OADH inhibition, and decreased levels of sirtuin 5 and sirtuin 3. The concerted perturbations in the brain acylation system 24 h after the OADH inhibition are thus revealed (Figure 9), that may underlie the long-term metabolic rearrangements (Figure 5).

## Discussion

Post-translational acylation of metabolic proteins and histones through covalent modifications of their lysine residue is an important mechanism contributing to organismal homeostasis and its changes in different pathologies (58–61). The major attention in this regard receives the most abundant modification of lysine residues—acetylation (62–65), whereas significantly less is known about the (patho)physiological role of other types of the acylation (66, 67). In particular, studies of the lysine glutarylation are mostly limited to pathological situations, e.g., when pathogenic mutations in glutaryl-CoA dehydrogenase cause increased levels of the glutarylating agent glutaryl-CoA (27). Our current result on an elevation in protein glutarylation in the animal brain affected by severe SCI, adds to results from independent studies on enhanced abundance of this modification in different pathologies, e.g., in patients with acute myocardial infarction (68), or upon impaired function of deglutarylase sirtuin 5 (69, 70), the enzyme known to be neuroprotective (25, 26).

In general, tissue level of protein glutarylation depends on the availability of glutaryl-CoA as a source of glutaryl residues, and activity of deglutarylase sirtuin 5. As a producer of glutaryl-CoA, OADH may thus have specific significance for the protein glutarylation. Our results about dependence of the brain protein glutarylation and/or sirtuin 5 expression on administration of the OADH-directed inhibitor TMAP, demonstrate tight connections between the OADH function and glutarylation in the rat brain. The OADH role in regulatory glutarylation is in good accord with the predicted nuclear localization signal of the enzyme (2). In addition to the known mitochondrial localization, the dual localization of the enzyme is also supported by experimental assays of OADH in the extramitochondrial fraction (41). In this regard, the known nuclear localization of the second component of the multienzyme OADH complex (*DLST*-encoded dihydrolipoamide transsuccinylase, EC 2.3.1.61) (71), that is common for the OADH and OGDH complexes, further supports the OADHC role in nuclear production of glutaryl-CoA. Thus, participating in the mitochondrial steps of the lysine and tryptophan catabolism, OADHC may also contribute to glutarylation of proteins, including those in nucleus, e.g., histones. In our samples, glutarylation of the low molecular mass proteins is negligible, compared to the major glutarylated protein bands of 130 and 70 kDa, responding to the TMAP treatment. This is in line with an earlier comparative study of the lysine glutarylation, succinylation, and acetylation detectable by specific antibodies in the brain cortex homogenates: Only the acetylation band is visible in the region of the protein molecular masses below 20 kDa (72). Hence, further studies are required for assessment of the OADH-dependent glutarylation of histones after their enrichment, not compatible with the experimental design of the current work. Nevertheless, nuclear function of OADH in protein glutarylation is in good accordance with our result on the long-term effects of the short-term perturbation of the OADH function by TMAP.

The short-term (24 h after the TMAP administration) inhibition of OADH by TMAP is evident from increased levels of the brain tryptophan (Figure 10). This metabolic indicator provides a good measure for the mitochondrial OADH activity that cannot be determined due to its overlap with that of OGDH. In this regard, it is also worth noting that the activity assayed *in vitro* is not equal to the substrate flux through an enzyme *in vivo*, as the flux depends on the *in vivo* concentrations of the enzyme substrates and regulators, which are not equal to those *in vitro*. Hence, the levels of related metabolites are better indicators of the *in vivo* fluxes than the levels of the enzymatic activities *in vitro*, assessing the enzyme functional expression. Simultaneously with the increased tryptophan level (Figure 10), the sirtuin 5 expression decreases (Figure 9), demonstrating an adaptation in protein deglutarylation to the decreased production of glutaryl-CoA by OADH. Interestingly, also the level of mitochondrial deacetylase sirtuin 3 decreases along with that of sirtuin 5. Apart from confirming the perturbations in the brain acylation system, this finding further links the OADH function to that of PDHC which produces acetylating residues in the form of acetyl-CoA. The lack of other short-term metabolic changes in response to TMAP agrees with the small flux through OADH in the brain, supporting the assumption that the delayed consequences of the TMAP action rely first of all on perturbations in the brain protein acylation.

Thus, the brain metabolic changes 8 weeks after the TMAP administration manifest a new metabolic state that is apparently controlled at epigenetic level by the short-term (patho)physiology-dependent decreases in the OADH function, linked to expression of sirtuin 5 and sirtuin 3. While sirtuin 3 controls mitochondrial metabolism where acetyl-CoA production by PDHC is of immense significance, extramitochondrial OADH may be involved in glutarylation of histones. Certain histones are known to be subject of glutarylation (66, 73)—the type of modification that changes the charge of lysine residues, thus regulating the chromatin state. For example, glutarylation of K91 of H4 histone is associated with higher transcriptional activity (74). Thus, the TMAP-induced changes in the glutarylation system involving the glutaryl-CoA producer OADHC and deglutarylase sirtuin 5, upon the short-term OADH inhibition may lead to the long-term consequences for gene expression due to the glutarylation-dependent chromatin remodeling. Obviously, this mechanism of metabolic regulation should depend on the chromatin state at the time of the TMAP action. This presumption is in good accord with conditional outcomes of the long-term TMAP effects, i.e., their dependence on (patho)physiological state of an organism. Remarkably, the difference between the LE and SCI rat brains in the reactivity of their glutarylation system to TMAP points to perturbed adaptability of SCI animals to metabolic challenges. After the TMAP treatment, the protein glutarylation does not increase in the brains of LE animals, as these animals are able to up-regulate their sirtuin 5. The upregulation is not possible in SCI animals, as their brain metabolism is significantly perturbed (26). As a result, these animals demonstrate increased glutarylation of the brain proteins in response to TMAP, compared to LE animals (Figure 7). A neuroprotective effect of sirtuin 5, observed in previous studies of SCI and other neuropathologies (25, 26), is in good accord with our current data, further supporting importance of the brain protein glutarylation for long-term systemic changes in neuropathologies. Dependence of these systemic changes on OADH inhibition, shown in this work, indicates that biphasic regulation of the OADH expression in spinal cord and skeletal muscles after SCI, known from independent transcriptomics studies (Supplementary Figure 1), is involved with the enzyme role in protein glutarylation that is of pathophysiological significance.

TMAP is a membrane-permeable trimethylated derivative of the true inhibitor, adipoyl phosphonate. Inside the cells, TMAP is hydrolyzed to adipoyl phosphonate by the action of intracellular esterases. Specific structural determinants in the active sites of OADH and OGDH are known, that promote preferential binding of AP to OADH, compared to OGDH preferring a shorter phosphonate analog, succinyl phosphonate (34). Our data on the TMAP-induced changes in the protein glutarylation along with the changed expression of OADH and sirtuin 5, are in good accordance with the specific action of TMAP on the brain OADH.

In addition to the TMAP-induced long-term changes in the protein components of the brain glutarylation system, associated with the changes in the level of protein glutarylation, some of the metabolic indicators of the long-term effects agree with independent data. One of the enzymes for which the functional significance of glutarylation is known, is carbamoyl phosphate synthetase 1 (CPS1, molecular mass 165 kDa, EC 6.3.4.16), producing carbamoyl phosphate for citrulline synthesis from ornithine. Glutarylation of CPS1 inhibits its function (23). Hence, the TMAP-induced decrease in citrulline simultaneously with increase in protein glutarylation in SCI vs. LE animals, are in good accordance with increased glutarylation of CPS1 as a molecular mechanism underlying the decreased citrulline level in the TMAP-treated SCI vs. LE animals.

The current study provides further evidence on functional differences between 130 and 70 kDa isoforms of OADH. Changed expression of the full-length 130 kDa isoform is grouped in the cluster 2 including the activity of a key protein of mitochondrial metabolism, OGDHC, and regulators of mitochondrial acylation—sirtuins 3 and 5 (cluster 2c, Figure 3). On the other hand, the N-terminal-truncated 70 kDa isoform (1) is clustered with activity of OADHCem (cluster 1b, Figure 3). Across all the treated animals groups, metabolic indicators of the two major clusters comprising either 70 (cluster 1) or 130 (cluster 2) kDa isoforms of OADH, undergo opposite changes, as compared to the levels in the control rats. Mostly, the coupled decreases in metabolic indicators occur in the 70-kDa-including cluster 1 (Figure 3), while the 130-kDa-including cluster 2 is associated with the increased levels vs. those in the control brains (Figure 3). This strong separation of the metabolic clusters associated with the OADH isoforms of 130 and 70 kDa points to the different biological roles of the isoforms.

It is worth noting that the most abundant fractions of glutarylated proteins in the brain cortex homogenates are those of 130 and 70 kDa. These molecular masses accurately match the apparent molecular masses of the previously characterized mammalian isoforms of OADH (26). Remarkably, in homogenates of liver, where the OADH expression is an order of magnitude higher than in the brain (34), the glutarylated proteins of the same molecular masses are stained by anti-glutaryllysine antibodies with a much higher intensity than in the brain homogenates (72). Moreover, the same protein bands are also detected as major glutarylated proteins in the fraction of liver mitochondria (72). After chemical glutarylation of the liver mitochondrial proteins, many additional protein bands become reactive to anti-glutaryl-lysine antibodies.

Autoglutarylation of OADH is supported by the coupled changes in the glutarylation of the brain proteins and expression of OADH isoforms, that is observed in the control and LE animals (Figure 7), and mass-spectrometric identification of glutarylation of recombinant human OADH (Figure 8). This side reaction of the catalytic process performed by 2-oxo acid dehydrogenases, is well-known from studies of other members of the enzyme family (53). Interestingly, one of the two glutarylated lysine residues of OADH, identified in the current work using eukaryotic expression of human OADH (K818, Figure 8), has been detected in the mouse liver enzyme under pathological conditions induced by knockout of glutaryl-CoA dehydrogenase (27).

Under pathological conditions, such as in the brain of SCI animals treated with TMAP, excessive glutarylation of the brain proteins is observed. This is accompanied by the TMAP-induced decline in the levels of the brain glutathione, localized to the same subcluster as PDHC activity. Association of this subcluster with the “blue” cluster 1 comprising glutarylation of 70 kDa proteins and expression of 70 kDa isoform of OADH, suggests that glutarylation of PDHC, the highly regulated system coupling the cytosolic and mitochondrial processes of glucose degradation, may mediate the systemic significance of OADH for insulin sensitivity, observed in independent studies (16–18, 75). The glutarylated residue of the PDHC component ODP2 is outside the enzyme active sites. Nevertheless, the glutarylation may affect the entry of CoA to the complex inner cavity, from where CoA arrives at the ODP2 active site. Thus, the position of the glutarylated ODP2 residue suggests a fine tuning of the PDHC function, probably involving conformational changes of the core, rather than a straightforward effect on the catalysis. One should also take into account that this residue of ODP2 and the neighboring residues are subject to other modifications, such as acetylation and ubiquitination. Hence, the observed effect of the ODP2 glutarylation level on the functional expression of PDHC in the brain homogenates may be due to complex regulation, potentially involving the protein stability. Functional significance of this modification of PDHC requires further studies in view of its potential role in the association between the OADH function and glucose homeostasis.

## Conclusion

The role of the *DHTKD1*-encoded OADH in the brain protein glutarylation, expression of sirtuins 3 and 5, and homeostasis of glutathione and amino acids is established. Glutarylation of ODP2 component of PDHC is shown, providing a molecular mechanism of the OADH association with systemic pathologies, such as diabetes.

## Data Availability Statement

The raw data supporting the conclusions of this article will be made available by the authors, without undue reservation.

## Ethics Statement

The animal study was reviewed and approved by the Bioethics Committee of Russian Cardiology Research and Production Complex and the Bioethics Committee of Lomonosov Moscow State University.

## Author Contributions

VB: conceptualization, writing—review and editing, supervision, project administration, and funding acquisition. AB, IK, AA, and VA: protein assays. AB and AT: purification of recombinant protein. TK: mass-spectrometry data acquisition. TK, VA, and LZ: analysis of mass-spectrometry data. AK: amino acid profiling. SR and AG: animal experiments. AB, AA, VA, and LZ: validation and formal analysis. VA and LZ: structural analysis. AB: writing—original draft preparation. AB, VA, LZ, and VB: visualization. All authors have read and agreed to the published version of the manuscript.

## Conflict of Interest

The authors declare that the research was conducted in the absence of any commercial or financial relationships that could be construed as a potential conflict of interest.

## Publisher’s Note

All claims expressed in this article are solely those of the authors and do not necessarily represent those of their affiliated organizations, or those of the publisher, the editors and the reviewers. Any product that may be evaluated in this article, or claim that may be made by its manufacturer, is not guaranteed or endorsed by the publisher.

## Footnotes

1
https://www.ncbi.nlm.nih.gov/geo/

2
https://rstudio.com

## Abbreviations

OADH: 2-oxoadipate dehydrogenase
OADHC: 2-oxoadipate dehydrogenase complex
OADHCem: extramitochondrial activity of OADHC
OGDH: 2-oxoglutarate dehydrogenase
OGDHC: 2-oxoglutarate dehydrogenase complex
PDHC: pyruvate dehydrogenase complex
DLAT: dihydrolipoamide acetyltransferase—PDHC E2 component
SCI: spinal cord injury
LE: laminectomy
TMAP: trimethyl adipoyl phosphonate
ThDP: thiamine diphosphate
GSSG: glutathione disulphide
MS: mass-spectrometry
NO ⋅: nitric oxide.
