# Supplementary material for: Delayed Impact of 2-Oxoadipate Dehydrogenase Inhibition on the Rat Brain Metabolism Is Linked to Protein Glutarylation
Source: Front Med (Lausanne). 2022 Jun 1;9:896263. doi: 10.3389/fmed.2022.896263 (PMC9198357; doi:10.3389/fmed.2022.896263)
Supplement: Supplementary file 1 [file Data_Sheet_1.docx]

Supplementary Material


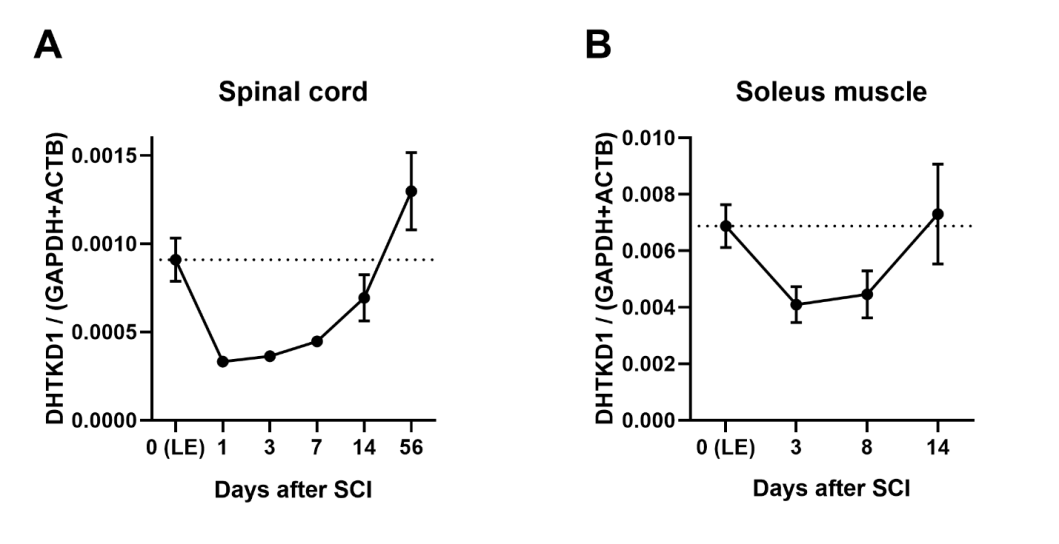


**Supplementary Figure S1. Changes in the DHTKD1 expression in spinal cord (A) and soleus muscle (B) upon spinal cord injury (SCI) in rats.** Data on mRNA abundance for the DHTKD1 gene are extracted from NCBI Gene Expression Omnibus database (datasets GSE45006 and GSE45550 ([1](#_ENREF_1)) for spinal cord and soleus muscle, respectively) and normalized to the sum of abundances for the GAPDH and ACTB genes. Dashed lines indicate the level of DHTKD1 expression in laminectomized (LE) animals.


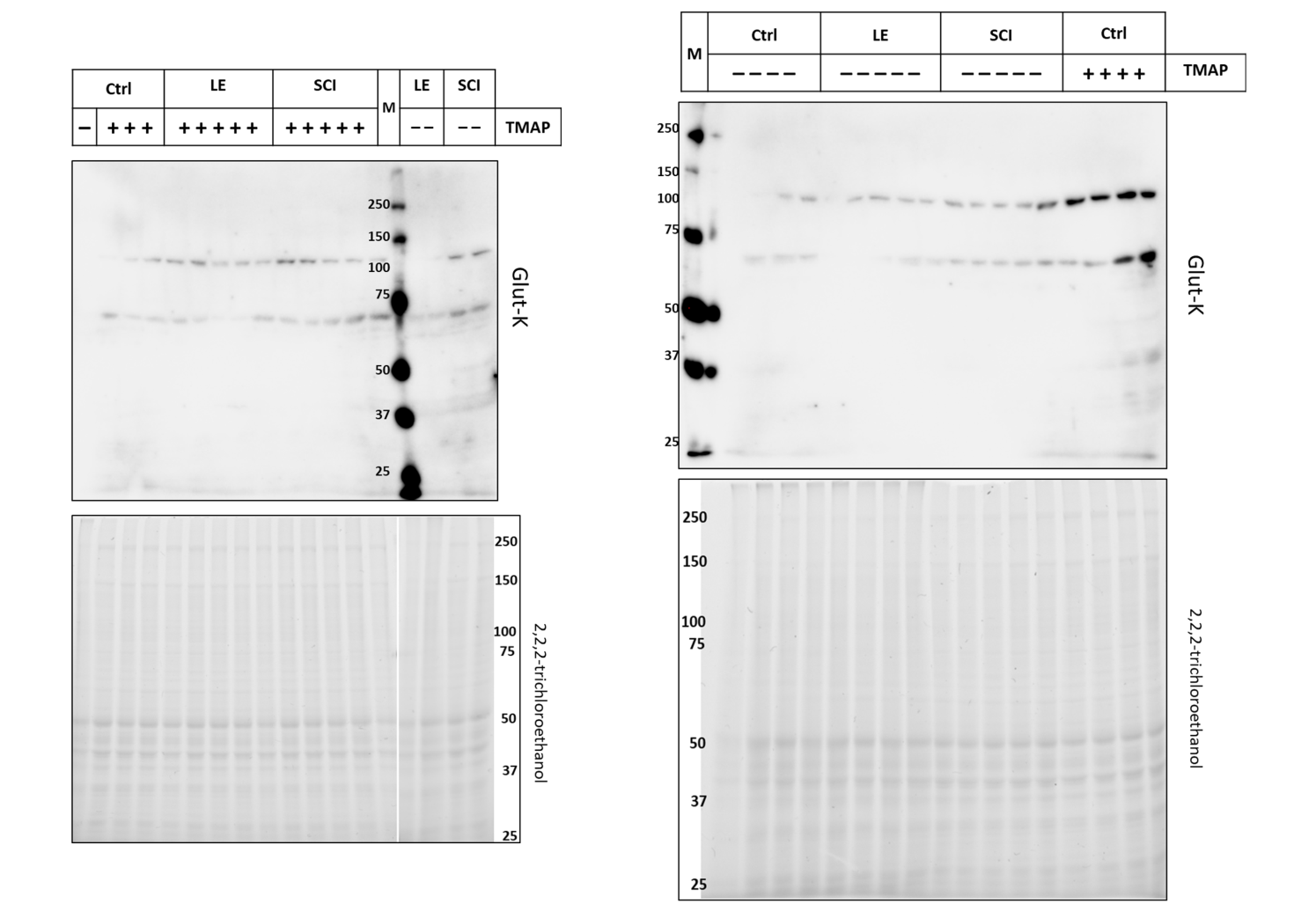


**Supplementary Figure S2. Western-blotting assessment of the brain levels of protein glutarylation eight weeks after administration of the OADH-directed inhibitor TMAP to the rats in different (patho)physiological states (control, LE and SCI).** Representative western-blotting results are shown above the images of the SDS-PAGE gels with the proteins stained by 2,2,2-trichloroethanol.

**
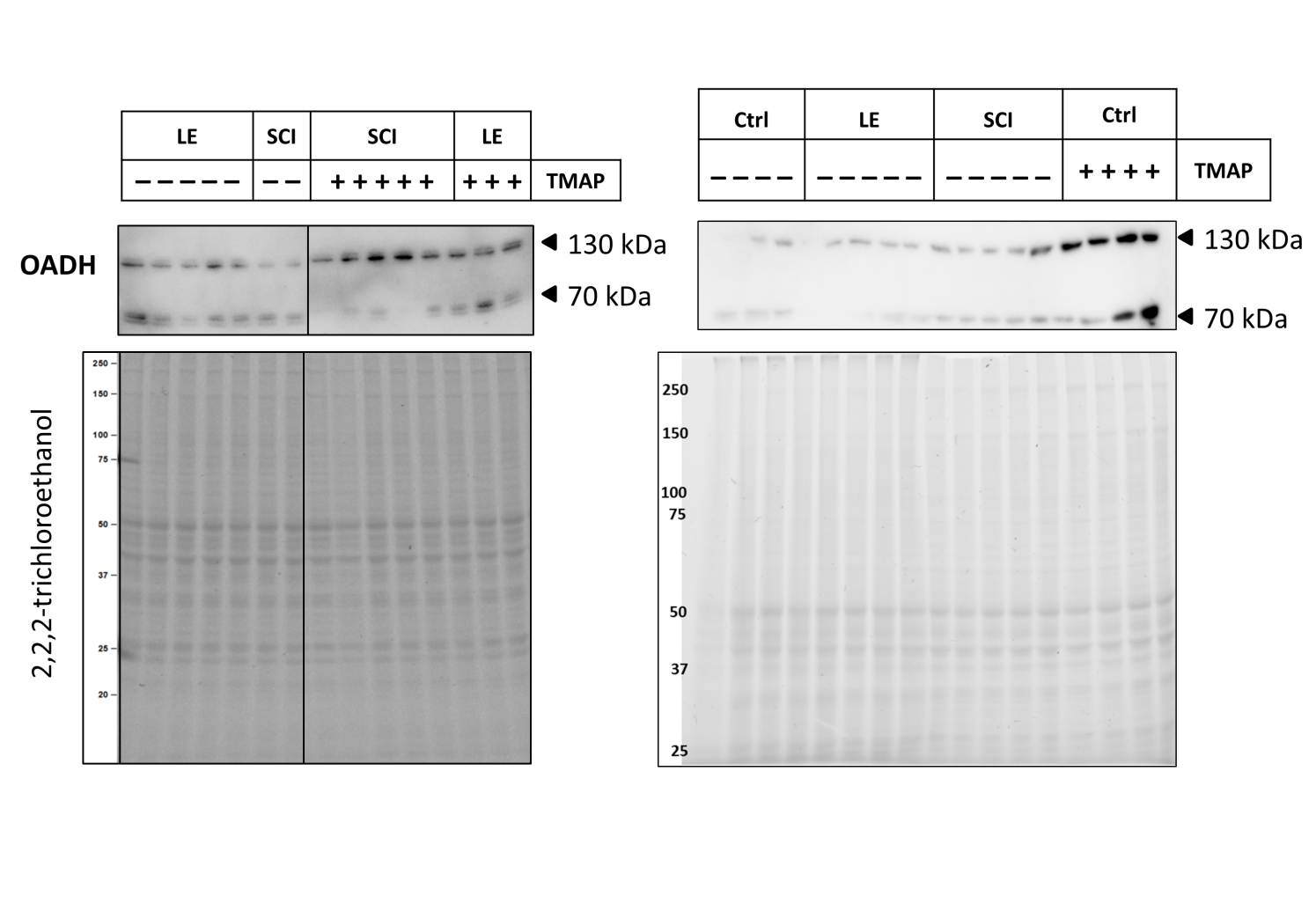
**

**Supplementary Figure S3. Western-blotting assessment of the brain expression of OADH eight weeks after administration of the OADH-directed inhibitor TMAP to the rats in different (patho)physiological states (control, LE and SCI).** Representative western-blotting results are shown above the images of the SDS-PAGE gels with the proteins stained by 2,2,2-trichloroethanol.


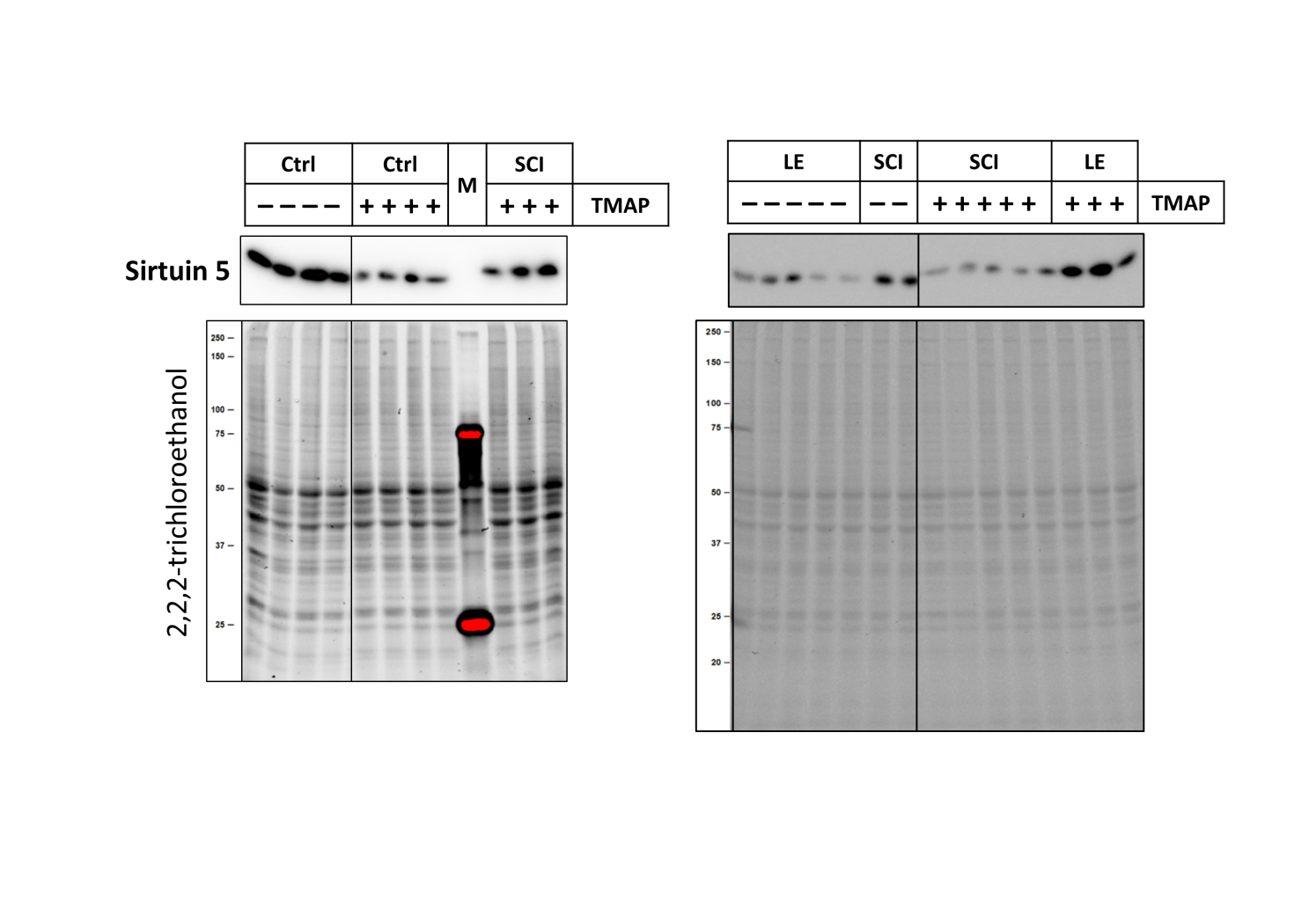


**Supplementary Figure S4. Western-blotting assessment of the brain expression of sirtuin 5 eight weeks after administration of the OADH-directed inhibitor TMAP to the rats in different (patho)physiological states (control, LE and SCI).** Representative western-blotting results are shown above the images of the SDS-PAGE gels with the proteins stained by 2,2,2-trichloroethanol.


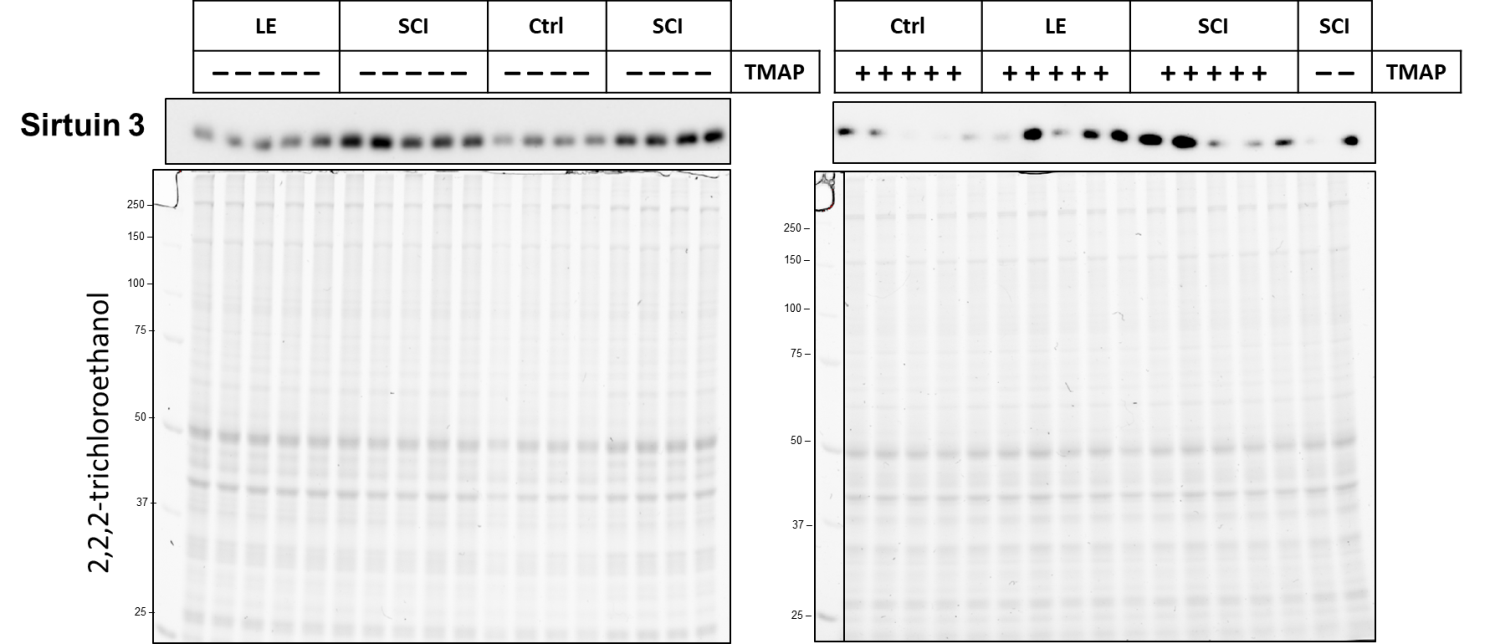


**Supplementary Figure S5. Western-blotting assessment of the brain expression of sirtuin 3 in rats eight weeks after SCI, or in groups with LE or control animals**. Representative western-blotting results are shown above the images of the SDS-PAGE gels with the proteins stained by 2,2,2-trichloroethanol.


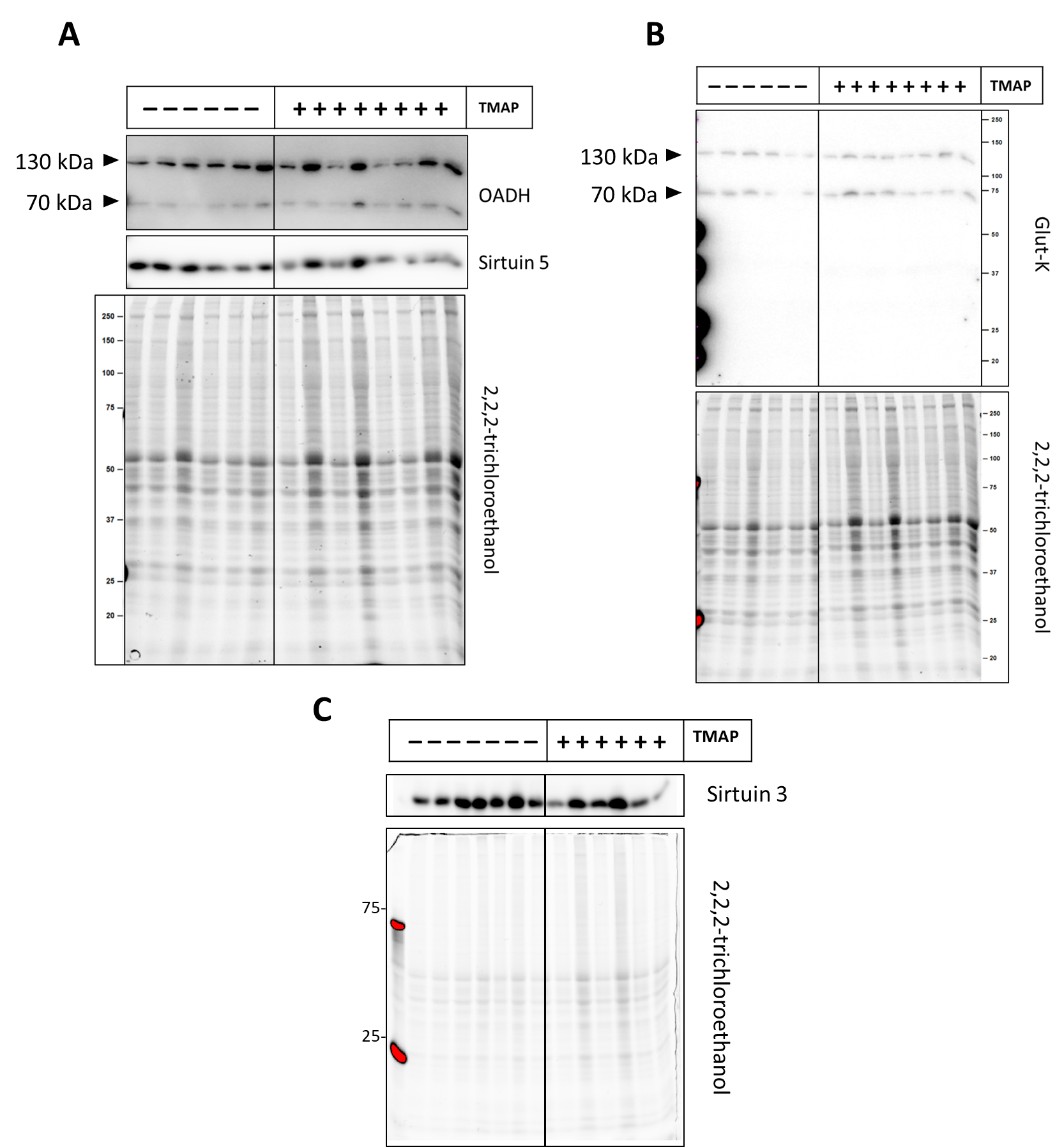


**Supplementary Figure S6. Western-blotting assessment of the brain expression of OADH, sirtuin 5 (A), protein glutarylation (B) and sirtuin 3 (C) 24 h after administration of the OADH-directed inhibitor TMAP to the control rats**. Representative western-blotting results are shown above the images of the SDS-PAGE gels with the proteins stained by 2,2,2-trichloroethanol.

**Supplementary References**

1. Baligand C, Chen YW, Ye F, Pandey SN, Lai SH, Liu M, et al. Transcriptional Pathways Associated with Skeletal Muscle Changes after Spinal Cord Injury and Treadmill Locomotor Training. BioMed research international. 2015;2015:387090.
